# Downregulation of ceramide synthase 1 promotes oral cancer through endoplasmic reticulum stress

**DOI:** 10.1038/s41368-021-00118-4

**Published:** 2021-03-22

**Authors:** Wen Chen, Chenzhou Wu, Yafei Chen, Yuhao Guo, Ling Qiu, Zhe Liu, Haibin Sun, Siyu Chen, Zijian An, Zhuoyuan Zhang, Yi Li, Longjiang Li

**Affiliations:** 1 State Key Laboratory of Oral Diseases & National Clinical Research Center for Oral Diseases & Department of Head and Neck Oncology, West China Hospital of Stomatology, Sichuan University, Chengdu, China; 2 Department of Oral and Maxillofacial-Head and Neck Oncology, Capital Medical University School of Stomatology, Beijing, China

**Keywords:** Oral cancer detection, Oral cancer detection

## Abstract

C18 ceramide plays an important role in the occurrence and development of oral squamous cell carcinoma. However, the function of ceramide synthase 1, a key enzyme in C18 ceramide synthesis, in oral squamous cell carcinoma is still unclear. The aim of our study was to investigate the relationship between ceramide synthase 1 and oral cancer. In this study, we found that the expression of ceramide synthase 1 was downregulated in oral cancer tissues and cell lines. In a mouse oral squamous cell carcinoma model induced by 4-nitroquinolin-1-oxide, ceramide synthase 1 knockout was associated with the severity of oral malignant transformation. Immunohistochemical studies showed significant upregulation of PCNA, MMP2, MMP9, and BCL2 expression and downregulation of BAX expression in the pathological hyperplastic area. In addition, ceramide synthase 1 knockdown promoted cell proliferation, migration, and invasion in vitro. Overexpression of CERS1 obtained the opposite effect. Ceramide synthase 1 knockdown caused endoplasmic reticulum stress and induced the VEGFA upregulation. Activating transcription factor 4 is responsible for ceramide synthase 1 knockdown caused VEGFA transcriptional upregulation. In addition, mild endoplasmic reticulum stress caused by ceramide synthase 1 knockdown could induce cisplatin resistance. Taken together, our study suggests that ceramide synthase 1 is downregulated in oral cancer and promotes the aggressiveness of oral squamous cell carcinoma and chemotherapeutic drug resistance.

## Introduction

Oral cancer is one of the most common malignant tumors in the head and neck region, and oral squamous cell carcinoma (OSCC) is the most common pathological type of oral cancer.^1^ Approximately one hundred thousand new OSCC patients are diagnosed worldwide every year.^2^ The main treatment for OSCC is combination therapy, including surgery, radiotherapy, and chemotherapy. The 5-year survival rate of early-stage patients is ~55%–60%, while the rate of late-stage patients is only 30%–40%.^3,4^ The overall survival of OSCC patients has not changed significantly in the last few decades.^5^ Therefore, it is necessary to analyze the molecular mechanisms of the development of oral cancer and strive for a new breakthrough in the study of OSCC diagnosis and treatment.

Ceramide (CER) is a kind of sphingolipid with hydrophobic chains that participates in multiple physiological functions. CER is synthesized by six different ceramide synthases (CERS). CERS are located on the endoplasmic reticulum (ER) and are necessary for the synthesis of CER. Each CERS has different selectivity for the synthesis of endogenous CER with different fatty acid chain lengths.^6^ Treatment with exogenous CER promoted differentiation and inhibited proliferation in a squamous cell carcinoma cell line.^7^ In addition, the function of CERS in tumors has been increasingly studied in recent years. CERS can regulate cell apoptosis, cell cycle arrest, and cell senescence.

Previous studies have demonstrated that in OSCC, CERS are involved in the regulation of apoptosis,^8^ EGF receptor modulation, inhibition of neovascularization,^9^ and enhancement of the anticancer actions of chemotherapy agents.^10^ More importantly, Karahatay’s study showed that C18 CER was the only decreased CERs in human head and neck squamous cell carcinoma (HNSCC) tissues, and decreased C18 CER level was strongly correlated with higher overall stages of the primary HNSCC tumors.^11^

C18 CER is mainly synthesized by CERS1.^12^ Koybasi found in HNSCC cells, overexpression of CERS1 resulted in impaired cell growth, which was related to telomerase activity and mitochondrial dysfunction.^12^ Similarly, Senkal found that knock-down of CERS1 in HNSCC cells resulted in attenuation of apoptosis due to the repression of casepase-3 and caspase-9 activity.^13^Moreover, CERS1 is also linked to chemotherapy resistance. It has been consistently reported that knockdown of CERS1 significantly protected HNSCC cells from chemotherapeutic agents, including gemcitabine, doxorubicin, and cisplatin, induced apoptosis.^14,15^ In response to cisplatin, CERS1 localized to mitochondria and induced mitophagy to promote cell death.^15^

In the present study, we used a transgenic mouse model and OSCC cell lines to explore the functional role of CERS1 in OSCC. Our study showed that downregulation of CERS1 in OSCC tissues significantly correlated with poor prognosis. CERS1 loss of function significantly promoted OSCC occurrence and progression both in vivo and in vitro. CERS1 loss of function could induce endoplasmic reticulum stress (ER stress) to promote chemotherapy resistance.

## Results

### CERS1 is related to the clinicopathological features and overall survival of OSCC patients

Our previous studies found that CER plays a very important role in the occurrence and development of head and neck tumors.^16^ In addition, the expression of C18 CER was significantly related to oral cancer.^12^ Igarashi^17^ found that different CERS members exhibited a characteristic fatty acyl-CoA preference. CERS1, a transmembrane protein of the ER, catalyzes the biosynthesis of C18 CER.^18,19^ Therefore, the expression of CERS1 might also influence oral cancer.

**Fig. 1:**
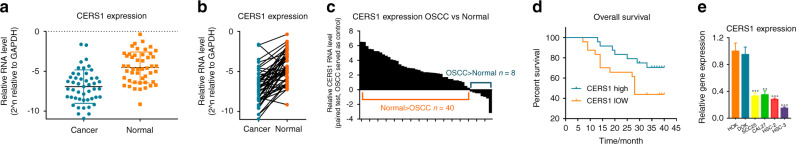
CERS1 correlated with OSCC and its prognosis. **a** RT-PCR was used to determine the expression of CERS1 in 48 OSCC tissues and 48 normal tissues. **b** RT-PCR analysis of the relative expression of CERS1 in 48 OSCC tissues and their paired adjacent normal tissues. **c** RT-PCR results showed that for most paired OSCC-normal tissues, CERS1 was downregulated in OSCC tissues. **d** Kaplan–Meier survival curves showed that low CERS1 expression was associated with poor overall survival. The log-rank test was used. **e** RT-PCR showed that the expression of CERS1 in OSCC cells (SCC25, CAL27, HSC-2, and HSC-3) was lower than that in non-malignant cells (HOK, DOK). For **a** and **e**, Student’s *t* test was used. For **b**, a paired *t* test was used. For **c**, in each paired tissue, the expression of CERS1 in OSCC were regarded as control, each column represented the fold expression of CERS1 in normal tissue in each paired tissue. Note: *P < 0.05; **P < 0.01; ***P < 0.001

To test this hypothesis, 48 patients with oral cancer were followed since 2016. Cancer tissues and para-cancer normal tissues were collected. By RT-PCR, we found that the expression of CERS1 in oral cancer tissues was lower than that in normal tissues (*P* < 0.001, Fig. 1a-c). In addition, the patients with high CERS1 expression survived longer (*P* = 0.049, Fig. 1d). The patients with lower CERS1 expression had a higher N stage (*P* = 0.085, Table 1), T stage (*P* = 0.043, Table 1), and overall clinical stage (*P* = 0.004). The expression of CERS1 in non-malignant cells, such as HOK and DOK, was higher than that in oral cancer cell lines, including SCC25, CAL27, HSC-2, and HSC-3 (*P* < 0.05, Fig. 1e).

**Table 1:** Analysis of CERS1 expression in tumor tissues of patients with OSCC

Characteristics	CERS1		*P* value
	Low expression	High expression	
Gender			
Male	14	16	0.561
Female	10	8	
Age/years			
≤59	13	13	1.000
>59	11	11	
Pathogenic site			
tongue	15	16	0.769
others	9	8	
Smoke			
no	13	8	0.152
yes	11	16	
Alcohol consumption			
no	14	8	0.085
yes	10	16	
T stage of tumor			
I–II	14	7	0.043*
III–IV	10	17	
N stage of tumor			
N0	21	12	0.004**
N1–2	3	12	
Clinical stage of tumor			
I–II	14	6	0.019*
III–IV	10	18	
Pathological grade of tumor			
I	14	17	0.376
II–III	10	7	

*P < 0.05; **P < 0.01

### CERS1 knockdown promoted oral cancer in vitro

**Fig. 2:**
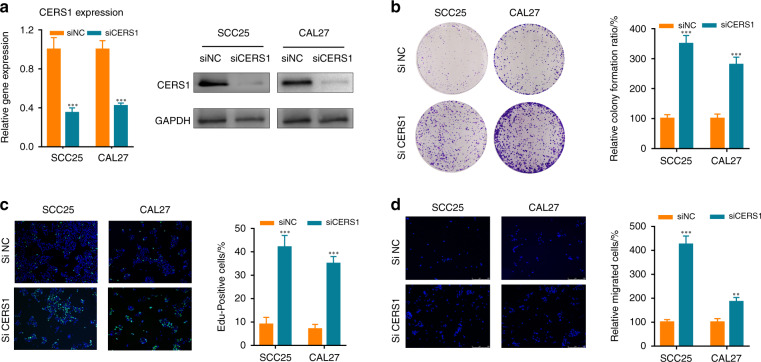
CERS1 knockdown promoted OSCC progression in vitro. **a** RT-PCR and Western blot confirmed CERS1 knockdown. **b**–**c** colony formation assay (**b**), and EdU incorporation assay (**c**) confirmed that CERS1 knockdown promoted OSCC proliferation. **d** transwell invasion assays confirmed that CERS1 knockdown promoted OSCC invasion ability. For **a**–**d**, Student’s *t* test was used. Note: *P < 0.05; **P < 0.01; ***P < 0.001

To verify the downregulation of CERS1 by siRNA interference in SCC25 and CAL27 cells, CERS1 expression levels were determined using RT-PCR and Western blot. As shown in Fig. 2a, CERS1 expression levels decreased to 35% and 42% after CERS1 knockdown.

Colony formation (Fig. 2b), EdU incorporation assays (Fig. 2c), and CCK-8 cell counting (Supplementary Fig. 2a) are common methods for cell proliferation detection. Results showed that CERS1 knockdown promoted proliferation in vitro. In addition, CERS1 knockdown promoted cell invasion (confirmed by the transwell invasion assay, Fig. 2d) and cell migration (indicated by the wound healing test, Supplementary Fig. 2b). In contrast, overexpression of CERS1 inhibited cell proliferation and invasion (Supplementary Fig. 1d-g).

### Cers1 knockout promoted OSCC occurrence in vivo

To elucidate the role of Cers1 in OSCC development, we first established a Cers1 knockout transgenetic C57BL/6 mouse model using CRISPR/Cas9 system. We deleted 238 bp around initiation codon in the first exon of Cers1. Cers1 gene and Gdf1 gene share the same exons and express bicistronic mRNA. Similar to the tansgenetic mice model established by Ginkel,^20^ the present Cers1 knockout strategy had no effect on the protein level of Gdf1 (Supplementary Fig. 1a). Also, in consist with Ginkel,^20^ we also observed that the Cers1−/− mice had exercise capacity defect and general tremor, which is more obvious with age.

**Fig. 3:**
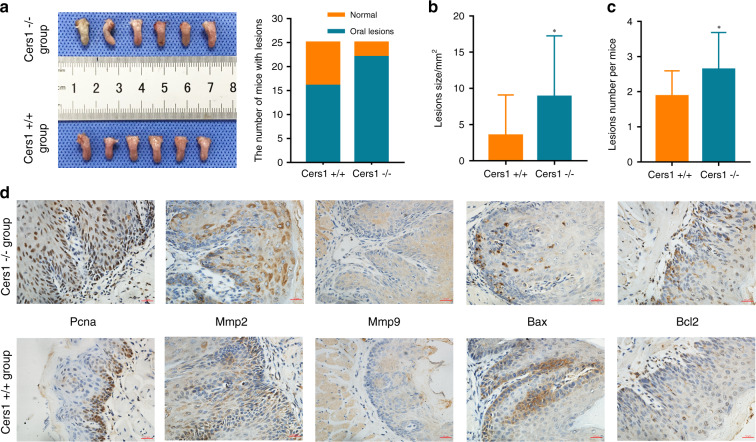
CERS1 knockout promoted OSCC occurrence in vivo. **a**–**c** gross appearances of the tongue are shown. The number of mice with lesions (**a**), lesion sizes (**b**) and lesion number per mouse (**c**) confirmed that CERS1 knockout promoted OSCC in vivo. **d** Immunohistochemical detection of hyperplastic lesions in tongue tissues. For (**b**) Student’s *t* test was used. For **c** rank-sum test was used. Note: *P < 0.05

An experimental model of 4NQO-induced OSCC was established as described previously.^21^ The consumption of 4NQO-water and basal diet per mouse of each group were comparable. Based on the gross appearance of the tongue, more mice developed obvious precancerous and cancerous lesions in the Cers1−/− group (22/25, 88%) than that in the Cers1 + /+ group (16/25, 64%) (Fig. 3a, Supplementary Fig. 1b). The average tumor lesion size in the Cers1−/− group was (7.82 ± 7.69) mm^2^. However, the average tumor lesion size in the Cers1 + /+ group was smaller at (3.5 ± 5.6) mm^2^ (*P* = 0.033, Fig. 3b). In addition, the number of lesions per mouse in the Cers1−/− group was significantly higher than that in the Cers1 + /+ group (2.23 ± 1.02 vs. 1.88 ± 0.72, *P* = 0.017, Fig. 3c). The results showed that knocking out Cers1 contributed to the formation of tongue lesions induced by 4NQO.

Histological examination of tongue tissues was conducted by two trained pathologists blinded to the sample identities. H&E staining showed that after treatment with 4NQO, the Cers1−/− mice exhibited different stages of oral carcinogenesis than the Cers1 + /+ mice. All samples were divided into four groups: normal epithelium, mild‑moderate dysplasia, severe dysplasia/carcinoma in situ, and carcinoma (Supplementary Fig. 2c). The results of the histopathological analysis of the Cers1−/− group and Cers1 + /+ group are shown in Table 2. A total of 36% (9/25) of mice in the Cers1−/− group developed tongue squamous cell carcinoma compared with 12% (3/25) of mice in the Cers1 + /+ group. These results indicated that Cers1 knockout enhanced 4NQO-induced tongue carcinogenesis.

**Table 2:** Histopathological examination of tongue lesions

Group	Mouse number	Mild-moderate dysplasia	Severe dysplasia or carcinoma in situ	Carcinoma	*P*
Cers1 + /+	25	19 (76%)	3 (12%)	3 (12%)	0.001
Cers1 −/−	25	6 (24%)	10 (40%)	9 (36%)	

(1) No specimen were categorized as normal epithelium; (2) the H&E results differed from gross appearance in Fig. 3a

Proliferating cell nuclear antigen (PCNA) is only present in normal proliferating cells and tumor cells and is closely related to the synthesis of DNA.^22^ It plays an important role in cell proliferation and is the key protein of abnormal cell proliferation.^23^ Immunohistochemical analysis demonstrated that PCNA was mainly expressed in the nucleus. By semiquantitative assessment of IHC staining, the rate of positive nuclear PCNA expression was found to be markedly high in the Cers1−/− group (*P* < 0.05, Fig. 3d, Supplementary Fig. 2d).

Matrix metalloproteinase-2 (Mmp2)^24^ and matrix metalloproteinase-9 (Mmp9)^25^ are important proteolytic enzymes that hydrolyze extracellular matrix and participate in the process of tumor growth and metastasis. To investigate cell invasiveness, Mmp2 and Mmp9 expression was assessed by immunohistochemistry. Mmp2 and Mmp9 were located in the cytoplasm. In the Cers1−/− group, high cytoplasmic expression of Mmp2 and Mmp9 was observed (*P* < 0.05, Fig. 3e, Supplementary Fig. 2d).

BAX, a member of the BCL2 family, is a core regulator of the intrinsic apoptosis pathway.^26^ BAX can affect the permeability of the outer mitochondrial membrane and subsequent initiation of the caspase cascade, which is considered a key step in apoptosis.^27^ The association of BAX with BCL2 has been demonstrated through coimmunoprecipitation assays.^28^ Immunohistochemistry results revealed that Bax and Bcl2 were localized predominantly in the cytoplasm. The expression levels of Bax in the Cers1−/− group were lower than those in the Cers1 + /+ group (*P* < 0.05, Fig. 3e, Supplementary Fig. 2d). In contrast, the expression of Bcl2 was higher (*P* < 0.05, Fig. 3e, Supplementary Fig. 2d).

### CERS1 knockdown increased the expression of ER stress markers and VEGFA

**Fig. 4:**
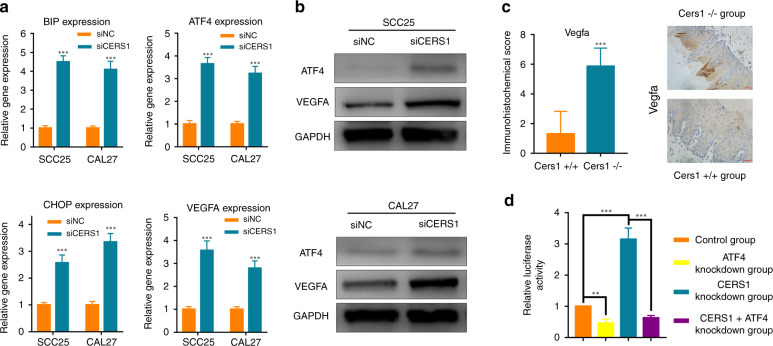
CERS1 knockdown induced ER stress, upregulating VEGFA. **a** and **b** RT-PCR and Western blot analysis showed the expression of ER stress markers (BIP, CHOP, ATF4) and VEGFA in OSCC cells. **c** Immunohistochemical detection showed the expression of VEGFA in mice with tongue tissue lesions. **d** Dual-luciferace results confirmed that CERS1 knockdown promoted VEGFA expression depended on ATF4. For (**a**) and (**d**) Student’s *t* test was used. For (**d**) rank-sum test was used. Note: **P < 0.01; ***P < 0.001

The ER is an important organelle for protein synthesis, folding, and secretion in eukaryotic cells. ER stress is a kind of cellular stress state caused by protein folding dysfunction of the endoplasmic reticulum that is induced endogenously or exogenously. The expression of ER stress markers, including binding immunoglobulin protein (BIP), ATF4, and C/EBP homologous protein (CHOP), was tested by RT-PCR and Western blot. BIP is a very important molecular chaperone in ER stress that can preferentially bind to misfolded proteins in the ER. The expression of BIP in CERS1 knockdown cells was higher than that in the control group (*P* < 0.05, Fig. 4a). ATF4 is involved in the regulation of many biological processes and plays an important role in ER stress.^13^ ATF4 was significantly upregulated after CERS1 knockdown (*P* < 0.05, Fig. 4a, b). ATF4 can bind to the CHOP promoter under ER stress conditions and induce the transcription of CHOP and related genes to promote the correct folding or degradation of residual proteins.^29^ Consistent with the expression of other ER stress markers, CHOP was also highly expressed in CERS1 knockdown cells (*P* < 0.05, Fig. 4a). RT-PCR of mouse lesion tongue tissues showed the same results (*P* < 0.05, Supplementary Fig. 2e).

Angiogenesis is a necessary process for tumor growth, invasion, and metastasis.^30^ Angiogenesis refers to the formation of new abnormal blood vessels in tumors, which provide nutrition and oxygen for tumor growth. Moreover, tumor cells secrete blood vessel growth promoting factor, which positively regulates this process.^31^ Vascular endothelial growth factor A (VEGFA) is an effective endothelial cell-specific regulator of angiogenesis that influences tumor growth and metastasis.^32^ It has been shown that ATF4 can regulate VEGFA transcription under ER stress in cancer cells.^33^ Our results found that Vegfa was located in the cytoplasm in mouse tongue specimens. Strong expression of Vegfa was observed in the Cers1−/− group mice (*P* < 0.05 Fig. 4c, Supplementary Fig. 2e). In addition, we found that VEGFA expression was higher in CERS1 knockdown cells in vitro (*P* < 0.05, Fig. 4a, b).

To prove that CERS1 knockdown led to increased VEGFA expression through ATF4, we used a luciferase reporter plasmid in which a VEGFA 5ʹ-flanking sequence (−2304 to +65 relative to the transcription initiation site) was fused to the firefly luciferase coding sequences in the pEZX-FR01 vector. The Renilla luciferase in the plasmid had its own replication origin (SV40), which was used as an internal control. The pEZX-VEGFA (Supplementary Fig. 2f) plasmid was cotransfected into CAL27 cells with different siRNAs. The activities of both firefly luciferase and Renilla luciferase were measured using the dual luciferase assay. As shown in Fig. 4d, a low level of VEGFA promoter activity was detected in ATF4 knockdown cells, whereas a high level of VEGFA promoter activity was detected in CERS1 knockdown cells. In addition, in both ATF4 and CERS1 knockdown cells, VEGFA promoter activity was also low. These results suggested that VEGFA promoter activity is responsible for the low expression of CERS1 through ATF4 upregulation.

### CERS1 knockdown led to mild ER stress causing cisplatin resistance

Cisplatin (referred to as DDP in the group name) is a first-line drug for OSCC treatment. The high incidence of drug resistance is the main limiting factor of the clinical efficacy of cisplatin.^34^ Recent studies have shown that high CERS1 expression renders cells more sensitive to cisplatin.^35^ As we have found that CERS1 is usually downregulated in OSCC samples (Fig. 1), we explored the relationship between CERS1 knockdown and cisplatin resistance.

A previous study showed that the ER adapts to endogenous and exogenous stressors by expanding its protein-folding capacity and by stimulating protective processes.^36^ Triggering continued mild ER stress to sustain ER homeostasis is considered a treatment for some diseases.^37^ Tunicamycin (TM), a UDP-N-acetylglucosamine-dolichol phosphate N-acetylglucosamine-1-phosphate transferase inhibitor, can block the initial step of glycoprotein biosynthesis in the ER. TM is now well known as a classical ER stress inducer.^38^ Mild ER stress can be induced by treating cells with low-dose TM (1 μg·mL^−1^, Sigma) for 2 h, which was used as a positive control.

**Fig. 5:**
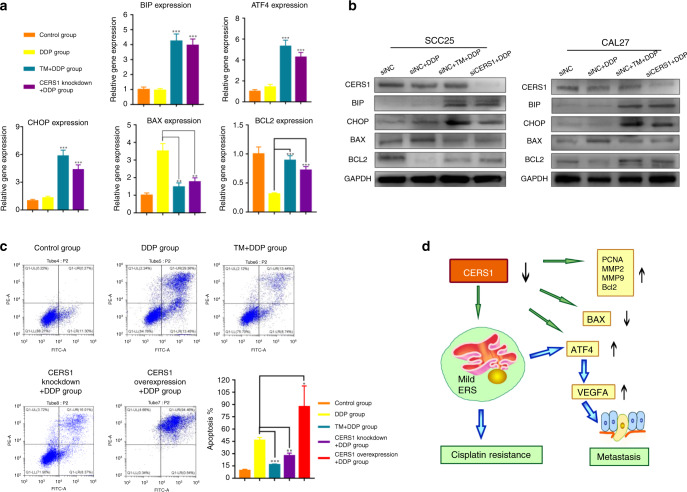
CERS1 knockdown regulated cisplatin resistance through mild ER stress. **a** and **b** RT-PCR (**a**) and Western blot (**b**) showed treated with low-dose TM and CERS1 knockdown had the same impact on OSCC cells for cisplatin resistance, that is, by inducing mild ER stress (the expression of BIP and CHOP was mildly upregulated) and suppressing apoptosis (the expression of BAX and BCL2). **c** Annexin V/PI double staining showed cisplatin could induce apoptosis; treated with TM or CERS1 knock-down contributed cisplatin resistance, whereas CERS1 overexpression resulted in sensitization to cisplatin. **d** the schematic diagram of present study. For **a** and **c** one-way ANOVA and Dunnett’s *t* test were used, “DDP group” was selected as the standard. Note: *P < 0.05; **P < 0.01; ***P < 0.001

There were four groups in the experiment that underwent various treatments as follows: siNC group, transfected with NC siRNA and treated with 1% DMSO; siNC+DDP group, transfected with NC siRNA and treated with cisplatin (40 nmol·L^−1^, Sigma) for 12 h; siNC+TM + DDP group, transfected with NC siRNA, pretreated with TM (1 μg·mL^−1^) for 2 h and then treated with cisplatin for 12 h; and siCERS1+DDP group, with CERS1 knockdown and then treated with cisplatin for 12 h. The expression of BIP, ATF4, and CHOP was higher in the siNC+TM + DDP and siCERS1+DDP groups than in the siNC and siNC+DDP groups (*P* < 0.05, Fig. 5a, b).

In addition, the annexin V/PI double staining assay (Fig. 5c, Supplementary Fig. 1h) showed cisplatin could significantly induce apoptosis. However, after treated with TM, the number of apoptotic cells decreased significantly. Similar to the anti-apoptosis effect of TM, knock-down of CERS1 significantly decreased the apoptosis ratio after DDP treatment. In contrast, overexpression of CERS1 resulted in sensitization to cisplatin. Also, cell viability test supported that knock-down of CERS1 resulted in cisplatin resistance, whereas overexpression of CERS1 obtained the opposite effect (Supplementary Fig. 1c). These findings indicated that knockdown of CERS1 could trigger mild ER stress and induce cisplatin resistance.

## Discussion

In this study, we investigated the roles and mechanisms of CERS1 in OSCC. Our data suggested that decreased levels of CERS1 might play important roles in oral cancer. CERS1 expression was lower in OSCC tissues than in controls. In addition, patients with lower expression of CERS1 in tumor tissues had a worse prognosis. Further data showed that downregulation of CERS1 resulted in the inhibition of cell apoptosis and promotion of cell proliferation and invasion, which involved the induction of mild ER stress and modulation of the protein kinase R-like endoplasmic reticulum kinase (PERK)/eukaryotic translation initiation factor 2α (eIF2α)/ATF4 pathway in OSCC. These results suggested that decreased levels of CERS1 conferred a growth advantage to cancer cells.

Our research group has performed this research with siRNA^14^ -mediated CERS1 knockdown in OSCC. In addition, knockout of Cers1 in mice was used to establish a mouse model of oral carcinogenesis. 4NQO is an aromatic amine heterocyclic compound that is often used as an inducer in oral cancer animal models.^39^ Oral cancer induced by the 4NQO carcinogen ranges from simple epithelial growth to invasive cancer. This process is similar to the real human disease process, making it more suitable to the capture and exploration of disease mechanisms.^40^ In this study, Cers1 knockout mice were treated with 4NQO. This was also the first study to explore the effect of Cers1 on the occurrence and development of oral cancer in vivo. Cers1 knockout mice were more likely to develop oral cancer, which was consistent with the vitro experiments and other studies.^41^

CERS are membrane proteins of the ER and synthesize (dihydro) CER with the N-acylation of the (dihydro) sphingosine backbone.^42^ CERS1–CERS6 have been identified, each of which catalyzes different lengths of acyl chains to produce CER. CERS1 uses C18-acyl-CoA, which is related to oral cancer cell autophagic cell death.^43^ CER can reduce protein kinase B (AKT) activity by activating protein phosphatase 2 A (PP2A), p38^44^ and protein kinase C (PKC),^45^ and then AKT reduces the phosphorylation level of BCL2.^44,46^ Finally, the decreased level of BCL2 and the ratio of BCL2 to BAX leads to cell death. Immunohistochemistry results confirmed this effect. Reducing CERS1 inhibits the synthesis of C18 CER, AKT, and BCL2 to inhibit apoptosis.

The ER is an organelle widely present in eukaryotic cells that regulates protein synthesis, folding, and aggregation after synthesis. An aggregation of misfolded proteins in the lumen and an imbalance of Ca2+ in the cytoplasm caused by various factors can lead to ER dysfunction, which can induce a series of related protein expression and cell phenotype changes, a condition called ER stress.^47^ ER stress mainly involves three ER transmembrane effector proteins, inositol-requiring enzyme 1, PERK, and activating transcription factor 6. When unfolded proteins accumulate, BIP dissociates from those proteins and binds to unfolded or misfolded proteins to help with proper folded.^48^ Activated PERK further phosphorylates the downstream eIF2α, ATF4, and CHOP.^49,50^ The expression of PERK-ATF4 has been positively correlated with VEGF.^51^ In our study, after knocking down/out CERS1, the expression of BIP, ATF4, CHOP, and VEGFA was higher than that in the control group. VEGFA promoter activity was related to CERS1 knockdown. However, ATF4 knockdown abolished this relationship. Therefore, downregulation of CERS1 promotes migration through ER stress, the PERK-ATF4 pathway and VEGFA.

Chemotherapy is one of the main treatments for oral cancer. However, some tumors become resistant during the course of treatment, which greatly limits the efficacy. Junxia Min’s research showed that CERS1 expression rendered cells more sensitive to cisplatin, carboplatin, doxorubicin, and vincristine.^35^ In addition, dasatinib induces apoptosis by upregulating the expression levels of CERS1.^52^ Different mechanisms of resistance to CERS1 have been investigated. However, we are far from having a full understanding of all CERS1 resistance-related pathways. As our study, CERS1 knockdown induced ER stress, which is a process that can remove misfolded proteins in the ER through the unfolded protein response to maintain ER homeostasis. If ER stress is not reversed, it will lead to cell function deterioration and cell death.^53^ However, mild ER stress sustains ER homeostasis, which is an attractive strategy for cancer.^54^ In our study, TM, a classical ER stress drug, was used as a positive control to explore the relationship between CERS1, mild ER stress, and drug resistance. Similar to the result of low-dose TM, CERS1 knockdown caused mild ER stress and then reduce the apoptosis caused by cisplatin. Overall, downregulation of CERS1 plays a negative role in chemotherapy for oral cancer (Fig. 5d).

## Materials and methods

### Cell culture and siRNA transfection

CAL27 cells, human normal oral keratinocytes (HOK), human dysplastic oral keratinocytes (DOK), HSC-2 cells, and HSC-3 cells were cultured in Dulbecco’s modified Eagle’s medium (DMEM) (Gibco, United States) and 10% fetal bovine serum (FBS) (Gibco, United States). SCC25 cells were cultured in Dulbecco’s modified Eagle’s medium/Nutrient mixture F-12 (DMEM/F12) (Gibco, United States) with 10% FBS and 400 μg·mL^−1^ hydrocortisone (Solarbio, China). All cells were cultured in a 5% CO_2_ incubator at 37 ^o^C. The CERS1 siRNA (GeneCopoeia, United States) target sequence was AAGGTCCTGTATGCCACCAGT. The ATF4 siRNA target sequence was CUGCUUACGUUGCCAUGAU. For control siRNA, the negative control siRNA from GeneCopoeia was used. CAL27 and SCC25 cells (6–8 × 10^5^) seeded in 6-well plates (Corning, United States) were transfected with 100 nmol·L^−1^ of each siRNA using Lipo2000 (Thermo Fisher, United States) and FBS-free medium. After 8 h of transfection, the medium was replaced with fresh FBS-containing medium. Cells were collected 48 h later.

### Cell proliferation assay

The cell counting kit-8 method (Dojindo, China) was used to determine cell proliferation and viability. CAL27 and SCC25 cells (5 × 10^3^) were plated in 96-well plates. After siRNA transfection, cells were incubated with 10% CCK-8 for an hour. Then, we used 480 λ to evaluate proliferation (microplate spectrophotometer, Sigma, United States).

The colony formation assay was also used to determine cell proliferation and viability. CAL27 and SCC25 cells (1 × 10^3^) were seeded in 6-well plates. After 10 days of growth, cells were fixed with 4% paraformaldehyde (Solarbio, China) for 20 min and stained with 0.2% crystal violet (Solarbio, China) for 5 min.

EdU DNA Proliferation in vitro Detection (GeneCopoeia, United States) was used to evaluate cell proliferation. Ten micromolar EdU in DMEM was used for CAL27 and SCC25 cells in 48-well plates (3 × 10^4^) for 2 h. After fixation and membrane permeabilization, iClick reaction buffer was used to detect EdU. Then, the cells were stained with DAPI (Solarbio, China) for 5 min. A Leica microscope was used for imaging (495 nm, 360 nm) and analysis.

### Cell migration and invasion assays

A wound healing test was used to test cell migration ability. CAL27 and SCC25 cells (1.5 × 10^6^) were seeded in 6-well plates. After cell adherence, we used 200 µL pipette tips (Thermo QSP, United States) to generate wounds. A Leica microscope was used for imaging at 0 and 48 h.

Cell invasion was tested by transwell invasion experiments. CAL27 and SCC25 cells (5 × 10^4^) were plated in Matrigel-coated transwell chambers (Corning, 8 μm) and cultured overnight. Then, the medium in the upper chamber was changed to FBS-free medium. The medium in the lower chamber was changed to 20% FBS medium. Twenty-four hours later, the cells that had moved across the membrane were fixed, permeabilized and counted by DAPI (Servicebio) staining. A Leica microscope was used for imaging (360 nm).

### Apoptosis assay

Annexin V/PI double staining (GeneCopoeia, United States) was used for apoptosis detection. CAL27 and SCC25 cells (1 × 10^6^) were collected. After labeling with Annexin V and propidine iodide, fluorescent dye solution was added to the cells away from light for 20 min. Then, flow cytometry (Beckman) was used for testing (488 λ and 560 λ), and FlowJo VX was used for calculation and analysis.

### Patients’ samples

Human cancer tissues and para-cancer (>1.5 cm from the tumor margin) normal tissues were collected from 48 OSCC patients in West China hospital of Stomatology, Sichuan University (China). After resection, the tissues were immediately frozen by liquid nitrogen and stored at −80 ^o^C for quantitative real-time PCR (RT-PCR). Written informed consent were signed by the patients. This study was approved by the Institutional Ethical Committee of West China hospital of Stomatology (WCHSIRB-OT-2016-047).

### Animal study

Both wild-type (Cers1 + /+) and Cers1 knockout (Cers1−/−) C57BL/N6 mice were obtained from VITALSTAR (Beijing, China). sgRNA for Cers1 knockout was: Cers1-gRNA1: atctgcgcataactcggcat ggg; Cers1-gRNA2: gggtggacagcgttgcgc tgg. The animals were housed in specific pathogen-free units at 24 ± 2 °C with 40%–60% humidity in a 14-hour light/10-hour dark cycle with freely accessible food at Sichuan University Animal Center (Chengdu, China). Six- to 8-week-old female mice (Cers1 + /+ C57BL/N6 mice, *n* = 25 and Cers1−/− C57BL/N6 mice, *n* = 25) were used for the experiments. A stock solution of 4NQO (Sigma, United States) was prepared at 5 mg·mL^−1^ (in propylene glycol). Two milliliters of stock solution was added to 100 ml of double distilled water to obtain a working concentration of 100 µg·mL^−1^. The mice were treated with 4NQO for 16 weeks and then observed for another 8 weeks. At the end of the experimental period, mice were sacrificed. Tongues were collected and then longitudinally bisected. The left half of the tongue was immediately fixed in 10% buffered formalin (Solarbio, China). The right half of the tongue was immediately put into RNAstore (Tiangen, China) and stored at −80 °C for RT-PCR. All animal experiments were approved by the Subcommittee on Research and Animal Care of Sichuan University (WCHSIRB-D-2017-227).

### Histopathological analysis

Tongue tissues from the Cers1−/− group and Cers1 + /+ group were processed for hematoxylin and eosin (H&E) staining. After 24 h of fixation in 10% buffered formalin at room temperature, the tongue tissues were embedded with paraffin. After paraffin sectioning, deparaffinization, and rehydration, the sections (4 μm) were stained with H&E (Solarbio, China). Histopathological diagnosis was performed by two experienced oral pathologists in a blinded manner. The tissues were classified into four types: normal epithelium, mild‑moderate dysplasia, severe dysplasia/carcinoma in situ, and carcinoma.

Immunohistochemical methods were used on sections (4 μm) of tongue tissues from hyperplastic lesions. The different groups of tongue tissues were deparaffinized and rehydrated in a graded ethanol series and distilled water. The slides were immersed in 0.01 mol·L^–1^ sodium citrate buffer (pH 6.0) and heated in a water bath at 95 °C for 30 min. Activities of endogenous peroxidases were inhibited by using 3% hydrogen peroxide. The sections were blocked with 3% BSA (Solarbio, China) for 20 min. Then, the sections were incubated overnight at 4 °C with anti-Bax antibody (1:1 000), anti-Bcl2 antibody (1:100), anti-Mmp2 antibody (1:1 000), anti-Mmp9 antibody (1:800), anti-Pcna antibody (1:500), and anti-Vegfa antibody (1:500). All antibodies for immunohistochemistry were from Servicebio (China). The slides were rinsed with PBS 3 times and incubated with biotinylated anti‑mouse/rabbit IgG (Servicebio, China) for 50 min at room temperature. Then, we used diaminobenzidine to visualize the slices. Finally, nuclei were counterstained with hematoxylin for 3 min at room temperature. PBS was used as a negative control instead of the primary antibody.

Five high power (40X) fields of each slides were randomly selected and evaluated under a light microscope (Leica, Germany). The assessment of IHC staining was performed by evaluating the staining intensity and the percentage of positive cells. The intensity was graded as 0 (no staining), 1 (mild staining), 2 (moderate staining), or 3 (strong staining). The proportion of stained cells was graded as 0 (negative), 1 (0–10% positive), 2 (10%–30% positive) or 3 (>30% positive). The index score ranged from 0 to 9.${\mathrm{Index}}\;{\mathrm{score}} = {\mathrm{percentage}}\;{\mathrm{of}}\;{\mathrm{staining}} \times {\mathrm{staining}}\;{\mathrm{intensity}}$

### Western blot

After treatment with siRNA, cells were collected, extracted with RIPA buffer (Beyotime, China), and boiled at 100 °C for 10 min. Then, the proteins were separated by sodium dodecyl sulfate polyacrylamide gel electrophoresis (SDS-PAGE) (10%, Bio-Rad, China) and transferred to PVDF membranes (0.22 μm, Millipore). After blocking with 5% BSA, the membrane was incubated with the primary antibody and then secondary antibody. Equivalent protein loading was confirmed using anti-GAPDH (#2118, Cell Signaling Technology, United States). Anti-CERS1 (#ab98062, Abcam, United States), anti-GDF1(#bs-1794R, Bioss, China) anti-ATF4 (#11815, Cell Signaling Technology, United States), anti-VEGFA (#ab46154, Abcam, United States), anti-BIP (#ER40402, Huabio, China), anti-CHOP (#5554, Cell Signaling Technology, United States), anti-BAX (#5023, Cell Signaling Technology, United States), and anti-BCL2 (#4223, Cell Signaling Technology, United States) were used. A chemifluorescence kit (Bio-Rad, China) and imaging system (Bio-Rad, China) were used for visualization of blots. ImageQuant 5.2 (GE Healthcare) was used for quantification.

### RNA extraction and quantitative real-time PCR

The cells were first treated with siRNA and then collected using RNAiso Plus (Takara, United States). After 20% volume of chloroform was added, and the cells were subjected to high-speed centrifugation, RNA was present in the aqueous phase. Then, an equivalent volume of isopropyl alcohol (Solarbio, China) promotes RNA degradation. After washing with 75% ethanol and dissolving in RNase-free water, RNA was transcribed into cDNA by a RevertAid RT Kit (Thermo, United States). Primers were designed by BLAST and synthesized by Sangon Biotech. SYBR Premix Ex Taq II (Takara, United States) and ABI Q7 were used for real-time PCR (RT-PCR). The primers for RT-PCR were listed Supplementary Table 1.

### Construction of VEGFA reporter plasmids

A 2.369 kb fragment containing a 5ʹ VEGFA sequence from −2304 to +65 relative to the transcription initiation site was amplified by PCR using Q5 High-Fidelity DNA Polymerase (NEB, United States). The forward primer with a SalI site was 5ʹ-GATGTCGACTTGCTGGGTACCACCATGGA-3ʹ, and the reverse primer, which had a XbaI site, was 5ʹ-GATTCTAGACAGAGCGCTGGTGCTAGCC-3ʹ. After digestion by QuickCut restriction endonucleases (Takara, United States), a DNA Ligation Kit (Takara, United States) was used to insert the PCR sequences into the SalI and XbaI sites of pEZX-FR01 (GeneCopoeia, United States), which contains a Rinella luciferase (Rluc) coding sequence with a CMV promoter and a promoter-less firefly luciferase (HLuc) coding sequence.

These recombinant plasmids were transfected into DH5α cells (TSINGKE, China) and amplified in nutrition agar plate (Solarbio, China) culture with kanamycin monosulfate (50 μg·mL^−1^, Solarbio, China) to select different monoclonal bacterial colonies. Then, the selected single clones were cultured in LB medium (Solarbio, China) with kanamycin. The recombinant plasmids were purified by a plasmid extraction kit (TIANGEN, China). All plasmid constructs were verified by direct sequencing (Sangon Biotech, Chengdu). The reporter plasmid was designated pEZX-VEGFA.

### Plasmid transfection and luciferase assay

CAL27 cells (3 × 10^4^) were plated in 96-well plates. After cell attachment, cells were transfected with the pEZX-VEGFA plasmid using Lipo2000 for 12 h. Then, transfection media was replaced with the appropriate growth media. Next, the cells were divided into four groups based on the different treatments: control group, siNC; ATF4 knockdown group, siATF4; CERS1 knockdown group, siCERS1; and CERS1 + ATF4 knockdown group, siCERS1 + siATF4. After transfection with siRNA, a Luc-Pair Duo-Luciferase HS Assay kit (GeneCopoeia, United States) and microplate spectrophotometer were used to determine the relative luciferase activity.

### Statistical analysis

IBM SPSS Statistics 20.0 was used for data statistics and analysis. Each experiment was performed independently at least three times with similar results. Data from one representative experiment are presented. The statistical methods are noted in the figure legends. *P* < 0.05 was deemed significant.

## Supplementary information

Supplementary table 1

Supplementary figure 1

Supplementary figure 2

## Data Availability

All data associated with this study are presented in the paper.
