# Supplementary material for: Downregulation of ceramide synthase 1 promotes oral cancer through endoplasmic reticulum stress
Source: Int J Oral Sci. 2021 Mar 22;13:10. doi: 10.1038/s41368-021-00118-4 (PMC7985500; doi:10.1038/s41368-021-00118-4)
Supplement: Supplementary file 1 — Supplementary table 1 [file 41368_2021_118_MOESM1_ESM.docx]

Supplementary table 1: Primer sequence for RT-PCR

|  | Forward Sequence 5’-3’ | Reverse Sequence 5’-3’ |
| --- | --- | --- |
| GAPDH | CTTTGGTATCGTGGAAGGACTC | GTAGAGGCAGGGATGATGTTCT |
| CERS1 | TGGTTCCTGTACATCGTGGC | CTCAGTGGCTTCTCGGCTTT |
| BIP | TTCTTGTTGGTGGCTCGACT | GTCAGCATCTTGGTGGCTTT |
| CHOP | GGAAACAGAGTGGTCATTCCC | CTGCTTGAGCCGTTCATTCTC |
| ATF4 | CCTTCACCTTCTTACAACCT | GTAGTCTGGCTTCCTATCTC |
| VEGFA | CTTGCCTTGCTGCTCTACCT | GCAGTAGCTGCGCTGATAGA |
| BAX | AACATGGAGCTGCAGAGGAT | CCAATGTCCAGCCCATGATG |
| BCL2 | GGTGGGGTCATGTGTGTGG | CGGTTCAGGTACTCAGTCATCC |
| Gapdh(house mouse) | AGGTCGGTGTGTGAACGGATTTG | TGTAGACCATGTAGTTGAGGTCA |
| Cers1 (house mouse) | CACCACACACATCTTTCGGC | GCGGGTCATGGAAGAAAGGA |
| Bip (house mouse) | TGTGTGTGAGACCAGAACCG | TAGGTGGTCCCCAAGTCGAT |
| Chop (house mouse) | GCAGCGACAGAGCCAGAATAA | ACCAGGTTCTGCTTTCAGGT |
| Atf4 (house mouse) | CCAGGGGTTCTGTCTTCCAC | AGAGCCCAGGTAGGACTCTG |
| Vegfa (house mouse) | AACTTCTGGGCTCTTCTCGC | CCAGCTCCGATCGGTTTGT |
